# Interspecific interactions change the outcome of sexual conflict over prehatching parental investment in the burying beetle *Nicrophorus vespilloides*

**DOI:** 10.1002/ece3.1795

**Published:** 2015-11-12

**Authors:** Ornela De Gasperin, Rebecca M. Kilner

**Affiliations:** ^1^ Department of ZoologyUniversity of CambridgeCambridgeCB2 3EJUK

**Keywords:** Interspecific interactions, *Nicrophorus*, phoresy, sexual conflict

## Abstract

Sexual conflict arises when the optimal reproductive strategy differs for males and females. It is associated with every reproductive stage, yet few studies have considered how the outcome may be changed by interactions with other species. Here, we show that phoretic mites *Poecilochirus carabi* change the outcome of sexual conflict over the supply of prehatching parental investment in the burying beetle *Nicrophorus vespilloides*. Burying beetles require a small dead vertebrate for reproduction, which they prepare by shaving it, rolling up the flesh, and burying it. When pairs were given a medium‐sized mouse to prepare (13–16 g), mites changed how the costs of reproduction were divided between the sexes, with males then sustaining greater costs than females. We found no equivalent difference when pairs prepared larger or smaller carcasses. Thus, our experiment shows that the outcome of sexual conflict over prehatching parental investment is changed by interactions with other species during reproduction.

## Introduction

Sexual conflict arises when the reproductive interests of males and females diverge, causing natural selection to act differently on genes expressed in males and females (Lessells 2006, 2012; Kokko and Jennions 2014). It underlies every stage of reproduction (Chapman 2006; Lessells 2006; Kokko and Jennions 2014) drives antagonistic co‐evolution between male and female traits (Chapman 2006; Lessells 2006; Kokko and Jennions 2014) and can lead to reproductive isolation and speciation (Arnqvist et al. 2000). Although there has been extensive work on sexual conflict, relatively few studies have considered how the outcome may be changed by ecological traits in general and by interactions with other species in particular. The exceptions include the theoretical suggestions that predators might change the intensity of sexual conflict (Magurran and Seghers 1994; Bonduriansky 2014), and an experiment showing that the distribution of food can change the outcome of conflict over mating (e.g. Fricke et al. 2010).

Here, we consider how interactions between species can change the outcome of sexual conflict over the supply of parental investment. Parental investment brings benefits to offspring but is associated with a marginal decline in direct parental fitness (Trivers 1972). Sexual conflict in this context centers on how these costs are divided between partners, with selection favoring the parent that takes the lesser share (Arnqvist and Rowe 2005; Houston et al. 2005; Lessells 2006; Kokko and Jennions 2014). A change in the outcome of sexual conflict may be detected within a single generation by searching for a change in the way in which the costs of care are divided between parents (see Kokko and Jennions 2014; Kilner et al. 2015). To determine whether interspecific interactions change sexual conflict over parental investment, we experimentally investigated how the costs of care are divided between the sexes, within a single round of reproduction. In our laboratory experiment, parents were either exposed to a second species when they bred, or they were bred in isolation from it. In addition, we varied the potential costs associated with parental care to test whether the magnitude of these costs separately, or additionally, affected sexual conflict over parental investment.

Our study focused on the burying beetle, *Nicrophorus vespilloides,* and its interactions with a phoretic mite, *Poecilochirus carabi,* that commonly associates with it. Burying beetles exhibit elaborate biparental care, which is centered on the body of a small dead vertebrate (Pukowski 1933). The burying beetle parents prepare the carcass for reproduction by removing any fur or feathers, rolling the flesh into a ball, smearing it with antimicrobial exudates, and burying it in a shallow grave (Cotter and Kilner 2010; Scott 1998). Eggs are laid nearby in the soil and newly hatched larvae crawl to the buried ball of flesh. The carcass thus becomes an edible nest, housing and nourishing the developing larvae, which are also fed directly by their parents. The carcass is also a food source for parents, which potentially allows them to recoup any costs of reproduction (Boncoraglio and Kilner 2012). Roughly 8 days after pairing, the larvae disperse away from the scant remains of the carcass to pupate in the soil, while their parents fly off in search of further reproductive opportunities (Pukowski 1933).

The life cycle of the mite is closely aligned with the reproductive activities of the burying beetle. Mites from the *P. carabi* species complex comprise several species that are morphologically similar (Brown and Wilson 1992; Schwarz and Müller 1992). We focused on the effect of *P. carabi* mites *sensu stricto* because this is the most common mite found on *N. vespilloides* beetles in nature (Schwarz et al. 1998). They are readily apparent because the deutonymphs (the phoretic stage) are very mobile, large, and aggregate on the beetle's head and thorax. These mites are not parasitic but rather use the burying beetle as a vector, riding on the beetle between carrion, upon which the mites also depend for reproduction (Schwarz and Müller 1992). Thus, mites and beetles are rivals for the food resource that parents use to recoup their costs of reproduction (Boncoraglio and Kilner 2012), and this potentially gives mites the capacity to change the outcome of sexual conflict over parental investment. When a beetle locates a carcass, the mites disembark, molt, mate, and reproduce on the carrion, living alongside the developing larvae on the carrion themselves (Schwarz and Müller 1992). When the parents leave the carcass at the end of reproduction, they carry with them the next generation of mites (Schwarz and Müller 1992). We know from our previous experiments that mites can affect male and female life span when breeding on carcasses of between 8 and 14 g, and can also influence the success of the brood (De Gasperin and Kilner 2015; De Gasperin et al. 2015). Specifically, they can reduce male life span, reduce the success of the brood, and enhance, reduce, or be neutral for female life span, depending on the size of her mate. However, these observations were made on beetles that completed reproduction. We do not yet know whether these changes in life span result from activities carried out before or after hatching, nor is it clear how they relate to the size of the carcass being prepared.

Previous studies of sexual conflict over parental investment have focused primarily on one aspect of posthatching care in particular, namely the provisioning of offspring (reviewed by Harrison et al. 2009; but see Benowitz et al. 2013; Head et al. 2014). We chose instead to focus on one aspect of prehatching parental care, the preparation of the carcass, because this allowed us to vary the potential costs associated with parental care. This we achieved by supplying pairs with small, medium, or large carcasses. Males put relatively more effort into carcass preparation than females (Smiseth and Moore 2004). Therefore, if mites increase the costs of carcass preparation, we predicted that this is more likely to affect males than females and that costs should be even greater after preparing a large carcass.

A major advantage of using the burying beetle to analyze sexual conflict over parental investment is that we can easily measure correlates of fitness that are associated with supplying parental care (see below). Thus, our study differs from most previous empirical work on sexual conflict over parental investment by focusing on fitness, rather than by quantifying behavior itself. The rationale underpinning our experimental approach is this: Behavioral traits associated with care cannot straightforwardly be mapped onto fitness (Sheldon, 2002; Harrison et al. 2009) – yet measures of fitness are key to documenting sexual conflict (Clutton‐Brock, 1991; Lessells 2006; Harrison et al. 2009). Behavioral observations might add flesh to our conclusions and interpretations, as might analyses of gene expression and hormone traits. But unpicking the detail of these mechanisms is tangential to the central aim of this study. By measuring changes in fitness associated with the supply of parental care, we focus directly on the evolutionary consequences of interactions between males and females during prenatal investment.

## Materials and Methods

### Maintenance of the beetle and mite colonies

We used the laboratory‐born descendants of wild beetles collected from wooded areas near Cambridge, in Cambridgeshire, UK. Beetles maintained in the laboratory did not carry mites and were kept inside plastic containers (12 cm × 8 cm × 2 cm) filled with moist soil, kept at 20°C and on a 16:8 light to dark cycle, and fed twice a week with minced beef. The mites were kept separately inside containers (17 cm × 12 cm × 6 cm) filled with moist soil and with a beetle. They were fed once a week with minced beef.

### Experimental design

The logic underlying our experiment involves three steps. (1) We manipulated the potential costs of care by introducing mites and/or by varying the size of the carcass, and then (2) measured how these fitness costs were then divided between the male and the female. We assumed that there was sexual conflict over the division of these costs in each experimental treatment. (3) We assessed whether the outcome of sexual conflict (i.e., the way in which costs were divided between the sexes) changed as a consequence of our manipulations.

1. Manipulating the fitness costs associated with supplying care

We manipulated the potential costs of parental care, and the opportunity to interact with mites during carcass preparation, using a fully factorial 3 × 2 experimental design. We gave pairs of virgin sexually mature beetles a dead mouse from one of three size classes to prepare: small (4–8 g), medium (13–16 g), or large (20–25 g): Each size category is well within the range of carcass sizes used in previous experiments on *N. vespilloides* (e.g., Eggert and Müller 1992, 1997). Half of the pairs in these treatments prepared the carcass in the absence of mites. For the remaining pairs, we added 10 deutonymphs (the phoretic stage of the mite) to the breeding boxes when we paired the beetles (in nature, a pair of beetles carries 8–16 deutonymphs between them at the start of the breeding season (Schwarz and Müller 1992)). We established 6 treatments (large carcass, no mites (*N *=* *22) or with mites (*N *=* *23); medium carcass, no mites (*N *=* *23) or with mites (*N *=* *22); small carcass, no mites (*N *=* *23) or with mites (*N *=* *21)), with treatments distributed over three experimental blocks. Fifty‐six hours after pairing (when the carcass is prepared, just before the larvae hatch), we stopped the breeding event. At this point, we described the extent of carcass preparation with a binary measure (1 = ‘fully prepared’, and 0 = ‘still bearing fur’). Description of the extent of carcass preparation was done blind to the mite treatment. At this stage, we also measured clutch size by counting all the eggs observable in the bottom of the breeding box. Previous observations of breeding events (without mites) in our laboratory have shown that this estimate of clutch size is highly correlated with the actual clutch size (Pearson's correlation *r *=* *0.908; M. Schrader unpubl. data).

We also removed each adult at this point (adults never carried mites at 56 h), placed it alone inside a small plastic box with soil, and stored it to measure its life span. We decided to remove parents at this point even if the carcass was not fully prepared because after 56 h of pairing larvae start to arrive to the carcass (personal observation), and we did not want to confound the costs of pre‐ and posthatching care. We stored the individual boxes in a haphazard sequence with respect to treatment, and thus, subsequent feeding was done blind to the treatment. We fed all the beetles with minced beef twice a week until they died, when we measured the pronotum width, a widely accepted and accurate proxy for adult size.

Measuring the fitness costs associated with supplying parental care

We used life span after carcass preparation to measure the fitness costs associated with parental care, because several aspects of the burying beetle's reproductive biology mean that a longer life span contributes directly to greater fecundity in each sex and is therefore a good proxy for fitness. In our previous laboratory experiments, we have shown that a longer life span enhances lifetime reproductive success in both sexes by affording a greater number of opportunities for reproduction (Ward et al. 2009; Cotter et al. 2010, 2011). These findings in the laboratory are likely to mimic the situation in nature for two good reasons. First, *N. vespilloides* is an opportunistic breeder because each sex is reliant on locating small carrion to produce offspring (Schwarz and Müller 1992; Scott 1998). The longer each sex lives, the more likely it is to find this key resource. In addition, males that are unsuccessful at locating carrion may attempt to attract females for mating by secreting pheromones (Eggert and Müller 1989; Eggert 1992). In nature, males commonly sire offspring without attending the carcass upon which larvae are raised (Müller et al. 2007). Therefore, the longer a male lives, the more likely he is to be successful at acquiring matings and thence further reproductive success.

Assessing the division of fitness costs associated with supplying parental care

The measure of particular interest here is not the absolute life span of each sex, nor the absolute magnitude of any change in the difference in life span between males and females. This is because an absolute change in life span is likely to contribute to fitness is different ways in each sex, which means that it cannot usefully be compared directly between the sexes. To bypass these difficulties, we focus instead on the sign of the difference in life span between males and females. If the sign of this difference changes as a result of our manipulations (of mites and/or carcass size), then we can conservatively conclude that the costs associated with supplying parental care have been divided differently between the sexes and therefore that the outcome of sexual conflict has correspondingly changed.

### Statistical analyses

We analyzed the data with the statistical program R (v. 3.0.2; R Development Core Team 2011), running general linear mixed models with the lmer function (lme4 package Bates et al. 2012). We included the carcass size class as a categorical variable with three levels and the presence/absence of mites as a categorical variable with two levels, the interaction between these factors, and the difference in size between members of each pair (male–female). We included as random factors the family of the male when analyzing male life span, and the family of the female when analyzing female life span, nested within the experimental block, to control for possibly having brothers or sisters across experimental treatments. After finding a significant effect of the interaction between the presence/absence of mites and the carcass size class on the life span of the male, we ran three separate models comparing the life span of the male for each carcass size class to better understand the separate effect of each treatment (Sokal and Rohlf 2012). We included as explanatory variables the presence/absence of mites, the difference in size between members of each pair (male–female), and the block as a random effect. There were no significant differences in the size of the male, the female, or the size difference between them across treatments (*P* values were above 0.1). We obtained *P* values using the ‘Anova’ function (car package, Fox and Weisberg 2011) with type III sum of squares, to obtain overall *P* values, and with the ‘summary’ function to obtain estimates.

To analyze the effect of the treatments on the division of costs between the sexes, we used exactly the same model, except that we used the difference in life span between members of each pair (male–female) as the response variable, and included as random effects the family of the male and of the female, both nested within the block. In this model, the interaction between the carcass size class and the mite treatment was marginally significant (*P = *0.09). We obtained *P* values using the ‘Anova’ function (car package, Fox and Weisberg 2011) with type III sum of squares, to obtain overall *P* values, and with the ‘summary’ function to obtain estimates. To better understand the separate effect of each treatment (Sokal and Rohlf 2012), we ran three separate models comparing the difference in life span between members of each pair for each carcass size class, including as an explanatory variable the presence/absence of mites, and the block as a random effect. We calculated the effect size (Cohen's *d*; Cohen 1992) of the presence of the mites on the difference in life span between each pair for each carcass size class, following the methodology described in Thalheimer and Cook 2002 (page 7), and using the *F*‐statistic obtained with the ‘anova’ function (stats package, Team, 2002).

We also compared the clutch size across treatments. We included the carcass size class as a categorical variable with three levels and the presence/absence of mites as a categorical variable with two levels, the interaction between these factors, and the absolute size of males and of females (as we know that female size strongly predicts clutch size). We included as random factor the family of the female nested within the experimental block. We obtained *P* values using the ‘Anova’ function (car package, Fox and Weisberg 2011) with type III sum of squares and with the ‘summary’ function (Bates et al. 2012). We checked the distribution of the residuals from our models to ensure they met the GLMM assumptions (Zuur et al. 2009).

Finally, we analyzed the extent of carcass preparation at 56 h after pairing with a generalized linear mixed effects model with a binomial distribution and included as explanatory variables the carcass size class, the presence/absence of mites, and the interaction between these factors. We included the block as a random effect. We obtained *P* values using the ‘Anova’ function (car package, Fox and Weisberg 2011) with type III sum of squares and with the ‘summary’ function (Bates et al. 2012).

## Results

### Effect on parental subsequent life span

**Figure 1:**
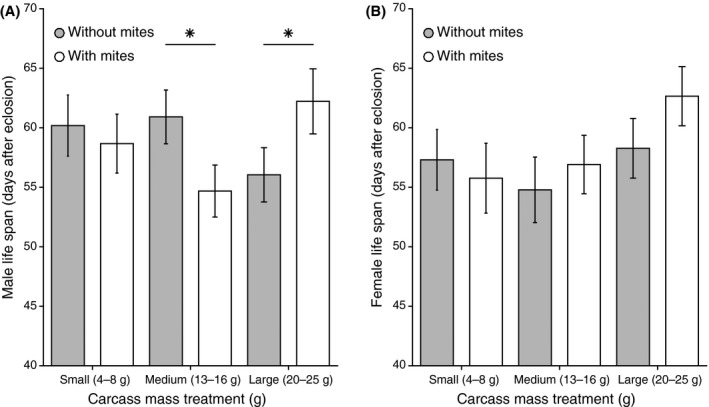
The effect of carcass size class and the presence/absence of mites on (A) Male life span and (B) Female life span. Means and SE are raw values, horizontal lines show significant differences. Mite's effect on male life span on medium carcasses: X21 = 5.96; *P* = 0.01; mite's effect on male life span on large carcasses: X21 = 4.47; *P* = 0.03.

For males, the size of the carcass influenced subsequent life span but the effect depended on whether or not mites were present (Tables 1, 2; Fig. 1A). To exclude the possibility that the effect on male life span was produced by the fact that most carcasses in the large carcass size class were not fully prepared by the end of the 56 h (see below), we ran a model where we included whether the carcasses had been fully prepared or not (as a categorical variable with two levels). This variable did not explain variation in the life span of the male (*P *=* *0.26), nor did it change the effect of the interaction between the carcass size class and the presence of the mites. The interaction between the presence of mites and the carcass size class had no effect on female life span (Tables 1, 2; Fig. 1B). When we analyzed the effects of mites separately for each category of carcass on male life span, we found that mites reduced male life span on medium carcasses (*χ* ^2^_1_ = 5.96; *P *=* *0.01; Fig. 1A), increased male life span on large carcasses (*χ* ^2^_1_ = 4.47; *P *=* *0.03; Fig. 1A) and had no effect on male life span on small carcasses (*χ* ^2^_1_ = 0.39; *P *=* *0.53; Fig. 1A).

**Table 1:** Results from the models analyzing the residual fitness of the parents using the Anova function (with type III SS). *N *=* *133 for male life span, *N *=* *134 for female life span and *N *=* *132 for difference in life span. Smaller sample sizes than those described in the methods are due to a couple of beetles escaping from their boxes prior to their death. Statistically significant results (*P *<* *0.05) are highlighted in bold

Explanatory variables	Male life span			Female life span			Difference in life span between the sexes		
	*χ* ^2^	df	*P* value	*χ* ^2^	df	*P* value	*χ* ^2^	df	*P* value
Mites	4.87	1	**0.02**	2.95	1	0.08	0.18	1	0.66
Carcass size treatment	3.52	2	0.17	1.69	2	0.42	4.41	2	0.10
Mites * Carcass size class	9.30	2	**0.009**	3.43	2	0.17	4.70	2	0.09
Size difference between the sexes	0.01	1	0.88	3.47	1	0.06	1.94	1	0.16

**Table 2:** Results from the models analyzing the residual fitness of the parents, using the ‘summary’ function*. N *=* *133 for male life span, *N *=* *134 for female life span, and *N *=* *132 for difference in life span. Statistically significant results (*P *<* *0.05) are highlighted in bold

Explanatory variables	Male life span				
	Estimate	SE	df	*t* value	*P*
Carcass size treatment medium	5.24	2.97	118.19	1.76	0.08
Carcass size treatment small	4.35	3.00	118.11	1.45	0.14
Mites	6.54	2.96	112.81	2.20	**0.029**
Size difference between the sexes	−0.21	1.55	118.93	−0.14	0.88
Carcass size treatment medium*mites present	−12.56	4.18	115.20	−3.00	**0.003**
Carcass size treatment small*mites present	−8.23	4.19	112.80	−1.96	0.052
	Female life span				
Carcass size treatment medium	−2.84	3.02	117.15	−0.94	0.34
Carcass size treatment small	0.81	3.05	118.52	0.26	0.78
Mites	5.19	3.01	117.78	1.71	0.08
Size difference between the sexes	2.92	1.57	121.00	1.86	0.06
Carcass size treatment medium * mites present	−2.04	4.22	116.55	−0.48	0.62
Carcass size treatment small * mites present	−7.59	4.24	117.98	−1.78	0.07
	Difference in life span between the sexes				
Carcass size treatment medium	8.29	3.96	118.61	2.09	**0.03**
Carcass size treatment small	3.74	4.00	120.02	0.93	0.35
Mites	1.69	3.95	119.28	0.42	0.66
Size difference between the sexes	−2.85	2.04	122.92	−1.39	0.16
Carcass size treatment medium * mites present	−10.82	5.54	118.22	−1.93	0.053
Carcass size treatment small * mites present	−1.00	5.56	119.43	−0.18	0.85

**Figure 2:**
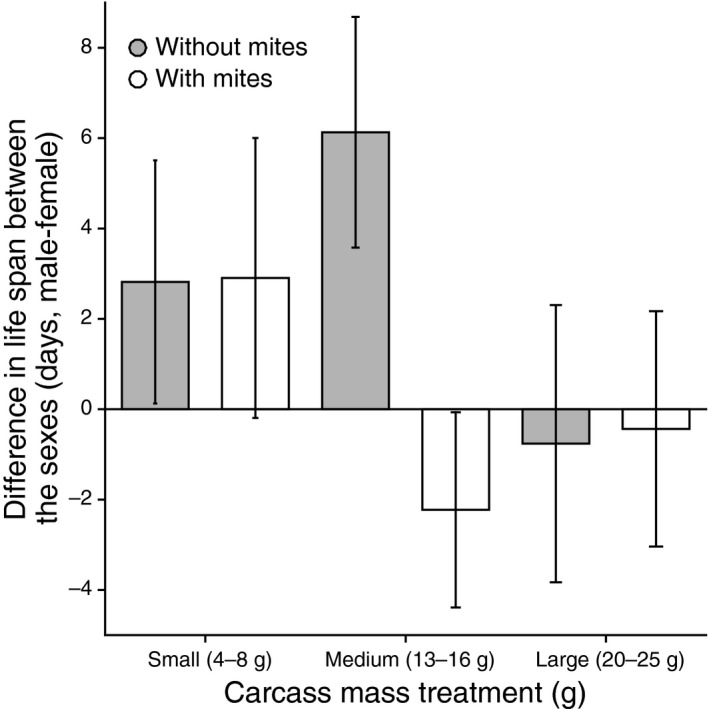
The effect of carcass size class and the presence/absence of mites on the difference in life span within each pair (male–female). Means and SE are raw values.

Using the sign of the difference in life span within a pair to assess the outcome of conflict, we found that mites changed the division of costs between parents, but that the effect depended on the size of the carcass they prepared together (Tables 3; Fig. 2). Splitting the dataset revealed that this effect was particularly pronounced on medium‐sized carcasses (Table 3; Fig. 2). Here, males subsequently lived longer than their partners when mites were absent, but females subsequently lived longer than their partners when mites were present (Table 3). The effect size of the mites' presence on the difference in life span on medium carcasses was large (Cohen's *d *=* *0.83).

**Table 3:** Results from the models analyzing the difference in life span between members of each pair for each carcass size class. *N = *22 for small carcasses without mites; *N *=* *21 for small carcasses with mites; *N *=* *23 for medium carcasses without mites; *N *=* *22 for medium carcasses with mites; *N *=* *21 for large carcasses without mites and *N *=* *23 for large carcasses with mites. Statistically significant results (*P *<* *0.05) and large effect sizes (Cohen's *d *>* *0.8) are highlighted in bold

Explanatory variable	SS	MS	Num df	Den df	*F*	*P*	Cohen's *d*
Small carcasses							
Presence of the mites	0.08	0.08	1	41	0.0004	0.98	0.006
Medium carcasses							
Presence of the mites	829.05	829.05	1	41.02	7.49	**0.009**	**0.83**
Large carcasses							
Presence of the mites	1.17	1.17	1	42	0.006	0.93	0.02

### Effect on clutch size

Neither the presence of the mites (*χ* ^2^_1_ = 1.58; *P *=* *0.20), nor the interaction between the carcass size class and the presence of the mites (*χ* ^2^_2_ = 2.70; *P *=* *0.25) influenced the size of the clutch. Female size strongly predicted clutch size (*χ* ^2^_1_ = 10.79; *P *=* *0.001), with larger females laying larger clutches (Estimate = 6.17; SE = 1.88; *t* value = 3.28; *P *=* *0.001), and carcass size class also influenced clutch size (*χ* ^2^_2_ = 46.43; *P *<* *0.0001). Using the ‘summary’ function, we found that clutches were smaller on medium (estimate = −5.31; SE = 1.63; *t* value = −3.24; *P = *0.001) and small carcasses (estimate = −11.22; SE = 1.64; *t* value = 3.28; *P < *0.00001) than on large carcasses.

### Effect on carcass preparation

Carcass size class significantly influenced the extent of carcass preparation (*χ* ^2^_2_ = 24.24; *P *<* *0.0001), though this time there was no interaction with the presence of the mites (*χ* ^2^_2_ = 1.39; *P *=* *0.49). Using the ‘summary’ function, we found that large carcasses were less likely to be completely prepared for reproduction within 56 h than either medium (Estimate = 2.03; SE = 0.53; *z* value = 3.77; *P *=* *0.0001) or small carcasses (Estimate = 2.68; SE = 0.63; *z* value = 4.24; *P < *0.00001). Nevertheless, irrespective of carcass size, mites accelerated carcass preparation (*χ* ^2^_1_ = 4.47; *P *=* *0.03).

## Discussion

Sexual conflict over parental investment centers on how the costs associated with parental care are divided between the sexes (Chapman 2006; Lessells 2006, 2012; Kilner et al. 2015). Here, we tested whether the division of the costs associated with prehatching care in the burying beetle varies according to interactions with a second species. We found no effect of mites on the size of the clutch laid by females. Instead, our results suggest that the costs of carcass preparation were divided differently between the sexes when mites were present, but that this effect was only evident when pairs were given medium‐sized carcasses (13–16 g) to prepare. Under these conditions, the sign of the difference between the sexes in subsequent life span was at its greatest (Fig. 2). Thus, our experiment shows that the outcome of sexual conflict over carcass preparation depends on interactions with two species: the size of the dead vertebrate used as a substrate for reproduction and the presence of phoretic mites from the *P. carabi* species complex that associate with the burying beetles. Our experiment also suggests that sexual conflict over parental investment originates in the prehatching phase of parental care and does not only result from provisioning offspring.

Although we have shown that the outcome of sexual conflict over prenatal investment is changed by the presence of mites, we do not know whether the outcome would have remained the same if we had allowed the beetles to complete reproduction. Perhaps mites increase the costs of posthatching care disproportionately for females, which may in turn vary with carcass mass. Thus, where females were favored during sexual conflict over prenatal investment, males might be favored during conflict over postnatal investment, yielding no net change in the outcome of sexual conflict that is driven by mites. Females may suffer postnatally from breeding alongside mites because they disperse with large numbers mites, or if they consume less of the carcass themselves. These possibilities would be worth investigating in further work.

An unexpected finding of our experiment was that the presence of mites accelerated the process of carcass preparation. Perhaps mites provoked faster carcass preparation because in this way, the beetles somehow limit carcass consumption by the mites. Alternatively, beetles may accelerate carcass preparation in order to disperse with fewer mites themselves at the end of the breeding event. We know from previous studies that males abandon the breeding event earlier when there are mites present, at least when breeding on a 8–12 g carcass (De Gasperin et al. 2015). Another explanation comes from the finding from field studies that the density of mites per beetle correlates strongly with the burying beetle population density (De Gasperin Quintero 2015). Perhaps the presence of the mites in our experiment was used as a cue by the beetles, indicating greater levels of intraspecific and intrageneric competition for resources, and thus beetles worked faster to conceal the carcass from potential rivals (Scott 1998). These explanations are not mutually exclusive, and careful experimental manipulations would be necessary to disentangle them: These tasks remain to be carried out in future work.

We hypothesized that a larger carcass would prove more costly to prepare, and predicted that males might bear the brunt of these extra costs. To some extent, this prediction was supported—at least when beetles bred in the absence of mites. In these circumstances, male life span was shorter having prepared a larger carcass, while female life span was broadly unaffected (Fig. 1). Why, though, did both males and females live longer after preparing a larger carcass in the presence of mites (Fig. 1)? We hypothesize that males and females may derive some fitness benefits from carcass preparation and that these may also vary with the size of the carcass. Our previous work suggests that both parents feed on the carcass during its preparation (Boncoraglio and Kilner 2012) and in so doing recoup their costs of reproduction (Boncoraglio and Kilner 2012; De Gasperin et al. 2015). Perhaps the net cost of reproduction is reduced when the extent of carcass preparation is more advanced. Since mites accelerated carcass preparation, perhaps both parents consumed more resources from these larger carcasses before the end of this treatment, and this contributed to their relatively long lives. On a medium carcass, perhaps mites exaggerated the net costs of carcass preparation for males, by accelerating the pace of carcass preparation and/or somehow preventing males from recouping their costs of carcass preparation. These possibilities remain to be investigated in future work.

Sexual conflict over parental investment is commonly analyzed using game theory and often without reference to wider ecological conditions (Chapman 2006; Lessells 2006). Though this approach has proved extremely insightful, our experiment reveals the limitations of applying these models to the real world, even under simplified conditions in the laboratory. Accordingly, future theoretical and experimental work on sexual conflict over parental investment could take greater account of interspecific interactions, the extent to which they fluctuate, and their capacity to favor one sex over the other.

## Conflict of Interest

We have no competing interests.
